# Effect of remote ischemic preconditioning on lung function after surgery under general anesthesia: a systematic review and meta-analysis

**DOI:** 10.1038/s41598-023-44833-w

**Published:** 2023-10-18

**Authors:** Shizuka Kashiwagi, Takahiro Mihara, Ayako Yokoi, Chisaki Yokoyama, Daisuke Nakajima, Takahisa Goto

**Affiliations:** 1 Department of Anesthesiology, Yokohama City University Graduate School of Medicine, Yokohama, Japan; 2 Department of Health Data Science, Yokohama City University Graduate School of Data Science, Yokohama, Japan; 3 Department of Anesthesiology and Intensive Care Medicine, Nagoya City University Graduate School of Medical Sciences, Nagoya, Japan; 4 Department of Anesthesia, Chiba Children’s Hospital, Chiba, Japan; 5 Department of Anesthesiology, Yokohama City University Medical Center, Yokohama City, Japan; 6 Department of Anesthesiology, Yokohama City University Hospital, 3-9 Fukuura, Kanazawa-Ku, Yokohama City, Kanagawa-Ken 236-0004 Japan

**Keywords:** Health care, Medical research

## Abstract

Remote ischemic preconditioning (RIPC) protects organs from ischemia–reperfusion injury. Recent trials showed that RIPC improved gas exchange in patients undergoing lung or cardiac surgery. We performed a systematic search to identify randomized controlled trials involving RIPC in surgery under general anesthesia. The primary outcome was the P_a_O_2_/F_I_O_2_ (P/F) ratio at 24 h after surgery. Secondary outcomes were A-a DO_2_, the respiratory index, duration of postoperative mechanical ventilation (MV), incidence of acute respiratory distress syndrome (ARDS), and serum cytokine levels. The analyses included 71 trials comprising 7854 patients. Patients with RIPC showed higher P/F ratio than controls (mean difference [MD] 36.6, 95% confidence interval (CI) 12.8 to 60.4, I^2^ = 69%). The cause of heterogeneity was not identified by the subgroup analysis. Similarly, A-a DO_2_ (MD 15.2, 95% CI − 29.7 to − 0.6, I^2^ = 87%) and respiratory index (MD − 0.17, 95% CI − 0.34 to − 0.01, I^2^ = 94%) were lower in the RIPC group. Additionally, the RIPC group was weaned from MV earlier (MD − 0.9 h, 95% CI − 1.4 to − 0.4, I^2^ = 78%). Furthermore, the incidence of ARDS was lower in the RIPC group (relative risk 0.73, 95% CI 0.60 to 0.89, I^2^ = 0%). Serum TNFα was lower in the RIPC group (SMD − 0.6, 95%CI − 1.0 to − 0.3 I^2^ = 87%). No significant difference was observed in interleukin-6, 8 and 10. Our meta-analysis suggested that RIPC improved oxygenation after surgery under general anesthesia.

**Clinical trial number:** This study protocol was registered in the University Hospital Medical Information Network (registration number: UMIN000030918), https://center6.umin.ac.jp/cgi-open-bin/ctr_e/ctr_view.cgi?recptno=R000035305.

## Introduction

Postoperative pulmonary complications (PPCs) are a major cause of mortality and morbidity after surgical operations. These complications include atelectasis, pneumonia, and the exacerbation of chronic lung disease. Among them, postoperative lung injury, including acute respiratory distress syndrome (ARDS), is one of the most serious complications. The etiology of PPCs includes inadequate smoking cessation, change of respiratory muscle function, postoperative upper airway obstruction due to residual anesthetics including neuromuscular blocking drug (NMBD), and postoperative pain that prevents patients from early mobilization. Advances in perioperative management made some of these factors modifiable by education on smoking cessation, lung protective ventilation, intraoperative monitoring of NMBD, and laparoscopic techniques with less postoperative pain. However, some part of surgical procedures induce ischemia–reperfusion injury and subsequent inflammatory responses. For example, in patients undergoing lung lobectomy, surgical manipulation and resection of the collapsed lung may cause lung injury. Additionally, re-expansion of the collapsed lung after one-lung ventilation induces ischemia–reperfusion injury, which subsequently triggers inflammatory responses in the contralateral lung as well as the local-side lung^1^. The use of cardiopulmonary bypass (CPB) during cardiovascular surgery also induces lung ischemia–reperfusion injury. During CPB, lung tissue suffers from loss of blood flow, which leads to hypoxia. Moreover, reperfusion of the ischemic lung after weaning from CPB can trigger complex pathophysiological situations, including that of ischemia–reperfusion injury^2^. Similarly, other types of surgery including bowel resection and vascular surgery often result in ischemia–reperfusion injury of the target organ. In addition to these surgery-specific factors, major surgery causes traumatic injury to the target organ, which leads to systemic inflammation and consequently injures distant organs such as the lung^3^. Perioperative fluid overload and increased vascular permeability will cause the collection of lung interstitial fluid, which leads to impaired diffusion capacity. These factors still remain unmodifiable risk of PPCs.

Remote ischemic preconditioning (RIPC) is a non-pharmacological approach to protect organs from ischemic events by the preliminary induction of a brief ischemia–reperfusion cycle of the limb. Although the underlying mechanism has not been fully elucidated, recent studies have implicated multiple molecular factors, including inflammatory cytokines, erythropoietin, bradykinin, adenosine, and hypoxia-induced factor α. Clinical research into RIPC started with a study of cardioprotection after myocardial infarction and subsequently focused on patients undergoing cardiac surgery^4^. Afterwards the effect of RIPC on other organs such as the kidney and on cognitive function was investigated^5,6^. Notably, some recent trials focused on respiratory function and showed that RIPC improved gas exchange in patients who underwent lung lobectomy or cardiac surgery^7–9^. Although a previous meta-analysis failed to show the pulmonary protection effect of RIPC^10^, a recent analysis reported by Zheng et al. observed that RIPC positively influenced clinical outcomes in patients undergoing cardiac surgery^11^. Several trials subsequently examined the lung protective effects of RIPC^12–14^. More recently, some studies reported the inhibitory effects of RIPC on inflammatory cytokines in patients undergoing major abdominal surgery^15–17^. The effect of RIPC on lung protective effect is needed to be clarified by a meta-analysis on updated data. We therefore performed an updated meta-analysis to investigate the respiratory effect of RIPC in surgical procedures performed under general anesthesia.

## Results

### Description of included trials

**Figure 1:**
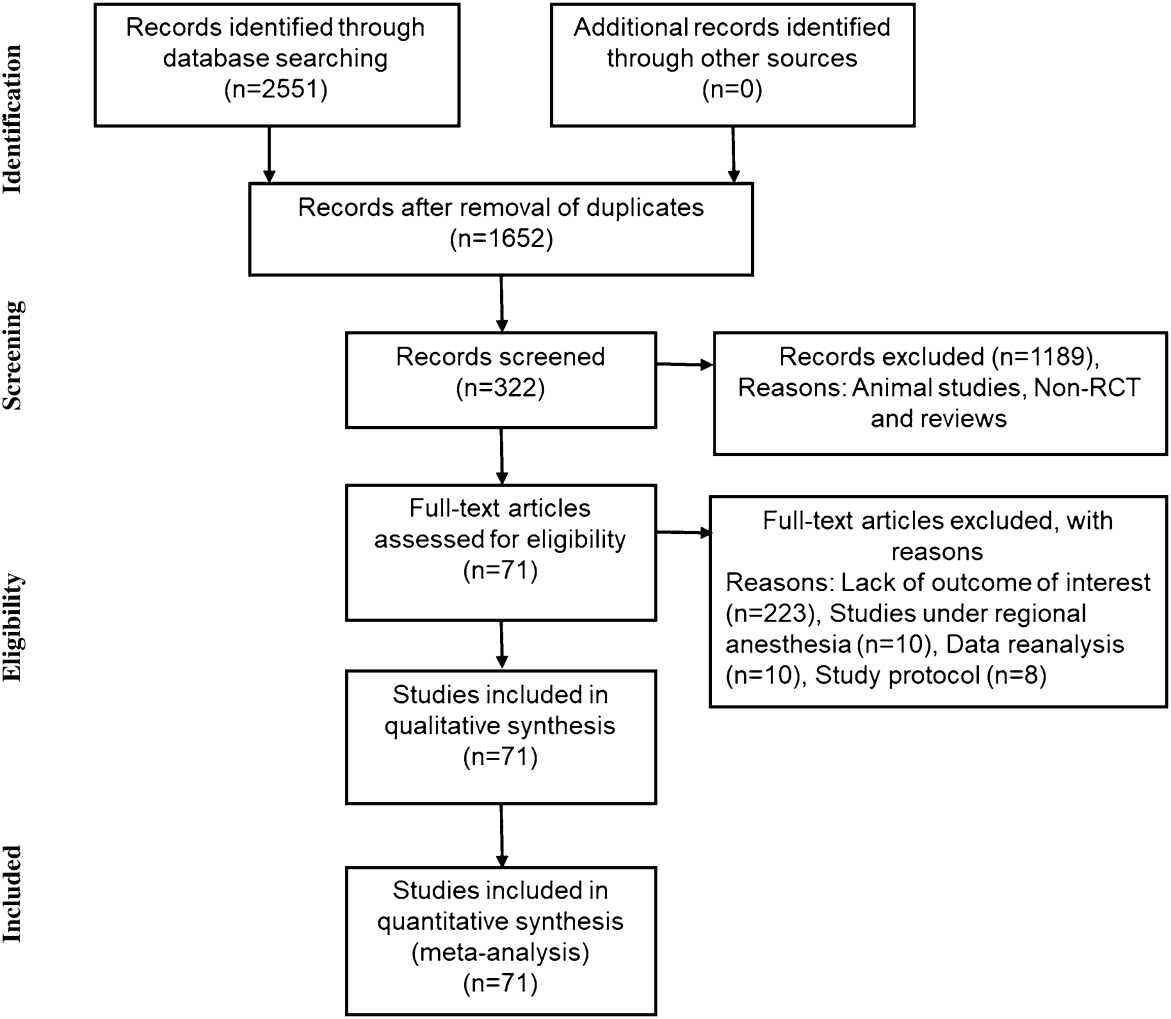
PRISMA flow diagram for study selection.

Through searching of the databases, 1652 unique publications were identified. After reviewing the titles and abstracts, 322 trials were retrieved for full-text evaluation. Among these, we excluded 249 trials mainly due to lack of the outcome of interest. Finally, 71 prospective RCTs were included in our quantitative analysis. The PRISMA flow diagram is shown in Fig. 1.

### Study characteristics

The features of the randomized studies included in this meta-analysis are listed in Supplemental Table S1. The 71 RCTs included 7854 patients: 3944 received RIPC and 3910 received a sham procedure. In 58 trials, the patients underwent cardiovascular surgery, whereas in 3 trials, the patients underwent lung lobectomy. The remaining trials included other types of surgery, such as major abdominal surgery, orthopaedic surgery, or transplantation surgery. Thirteen trials were conducted with patients under 18 years of age, most of whom underwent intracardiac repair^4,18–28^. Regarding the anesthetic agents, 17 trials used propofol for the maintenance of general anesthesia^8,9,15,16,29–40^, whereas the other trials used volatile anesthesia or did not restrict/document the anesthetic agent. In most cases, RIPC was performed after the induction of general anesthesia or before the start of surgery^5,7,15–17,19,21,23,24,27–32,35–37,39,41–66^; otherwise, it was performed before the start of cardiopulmonary bypass^4,8,13,20,67^ or 12–48 h before surgery^18,22,25,26,34^. A tourniquet was placed almost equally on the upper or lower limb. The RIPC protocols, in terms of the period of cuff occlusion and reperfusion were very similar: 3 cycles of 5 min of occlusion by inflation to 200–300 mm Hg and 5 min of reperfusion.

### Risk of bias

The quality of the 71 RCTs was assessed independently by two authors (SK and one of CY, AY, DN, or TM) using the Cochrane Collaboration tool. Detailed quality assessments are shown in Supplemental Table S2. Thirty-two of the 71 the included trials showed a ‘low’ risk of bias, whereas the rest were judged as ‘unclear’ as the methods used to protect against bias were not sufficiently reported.

### Effect of RIPC on oxygenation

**Figure 2:**
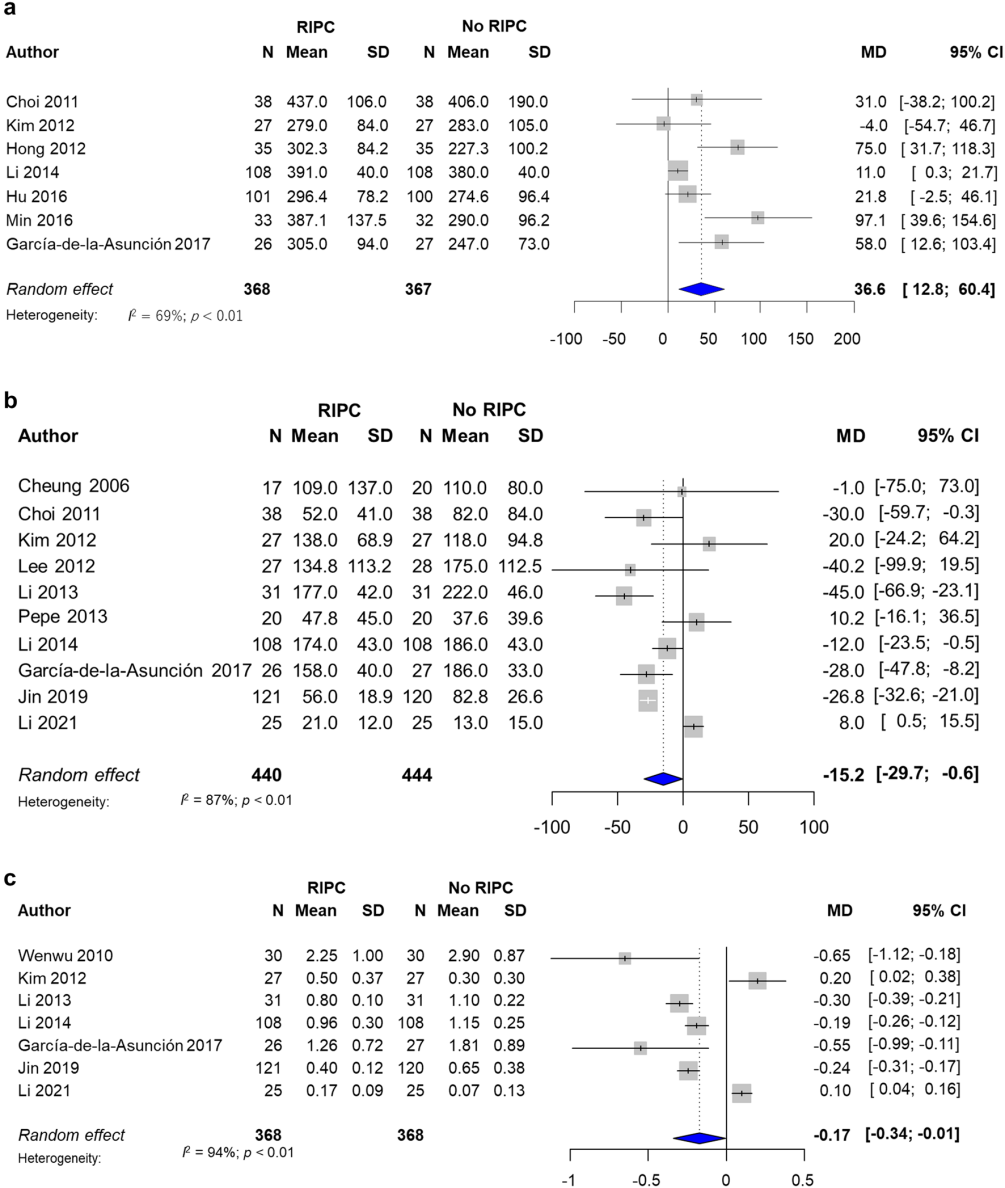
Forest plot with 95% confidence interval (CI) of P_a_O_2_/F_I_O_2_ (P/F) ratio (**a**), A-a DO_2_ (**b**), and respiratory index (**c**). *SD* standard deviation, *MD* mean difference.

Seven RCTs with 735 patients were analysed to investigate the effect of RIPC on the P/F ratio at 24 h after surgery^7–9,12,30,41,67^. The combined results are shown in Fig. 2a. In comparison to patients without ischemic preconditioning, those with RIPC showed a significantly higher P/F ratio at 24 h after surgery, with substantial heterogeneity (MD 36.6 Torr, 95% CI: 12.8 to 60.4, I^2^ = 69%) (Fig. 2a). When the analysis was repeated using trials with a low risk of bias (4 RCTs)^8,30,41,67^, we confirmed similar results (MD 50.4 Torr, 95% CI 4.9 to 96.0, I^2^ = 65%) (Supplemental Fig. S1). The evidence quality of the P/F ratio was graded as ‘low’ as there were limitations in terms of the presence of substantial heterogeneity and potential publication bias.

Our pre-specified subgroup analysis was performed from three perspectives: age (over 18 years old or not), anesthetic agent (total intravenous anesthesia or volatile anesthesia), and type of surgery (lung lobectomy or not). All seven of the RCTs targeted adult patients. Three RCTs were performed using total intravenous anesthesia^8,9,30^, and two RCTs were conducted in patients who underwent lung lobectomy^7,9^. No differences were found between the sub-groups (Supplemental Fig. S2). We additionally performed a *post-hoc* sub-group analysis according to the place of inflation cuff (arm or thigh) and total inflation time (over 15 min or not). The differences between subgroups were non-significant (Supplemental Fig. S2).

A-a DO_2_ and the respiratory index were also assessed. A-a DO_2_ at 24 h after the operation was assessed in 10 trials with 884 patients^4,7,9,13,14,21,24,29,41,67^, which indicated that A-a DO_2_ was significantly lower in the RIPC group, with significant heterogeneity (MD − 15.2 Torr, 95% CI − 29.7 to − 0.6, I^2^ = 87%) (Fig. 2b). Similarly to the P/F ratio and A-a DO_2_, RIPC was also associated with a lower respiratory index (MD − 0.17, 95% CI − 0.34 to − 0.01, I^2^ = 94%) (Fig. 2c)^7,9,13,14,18,29,41^.

### Effect of RIPC on MV period and ARDS

**Figure 3:**
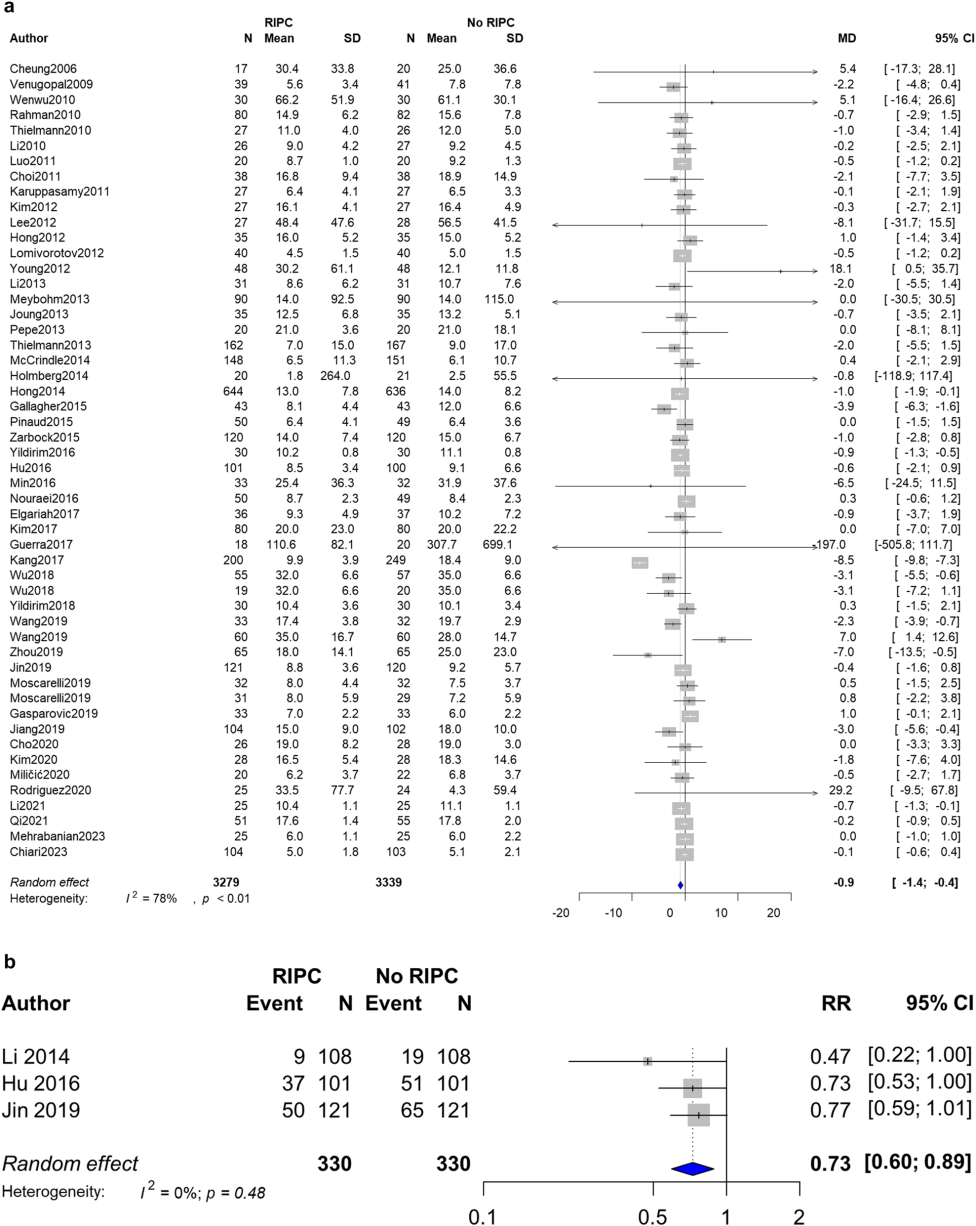
Forest plot with 95% confidence interval (CI) of the duration of mechanical ventilation (**a**) and the incidence of acute respiratory distress syndrome (**b**). *SD* standard deviation, *MD* mean difference, *RR* relative risk.

Fifty-two trials with 6,618 patients reported the duration of MV after surgery^4,5,8,12–14,18–21,24–37,39–41,43,44,47–57,59–62,65,67–69^, which showed that the RIPC group was weaned very slightly but significantly earlier from ventilation (MD − 0.9 h, 95% CI − 1.4 to − 0.4, I^2^ = 78%) (Fig. 3a). We detected no evidence of publication bias after a funnel plot analysis (Supplemental Fig. S3). Furthermore, the incidence of ARDS was lower in the RIPC group (RR 0.73, 95% CI 0.60 to 0.89, I^2^ = 0%) (Fig. 3b).

### Effect of RIPC on inflammatory cytokine levels

**Figure 4:**
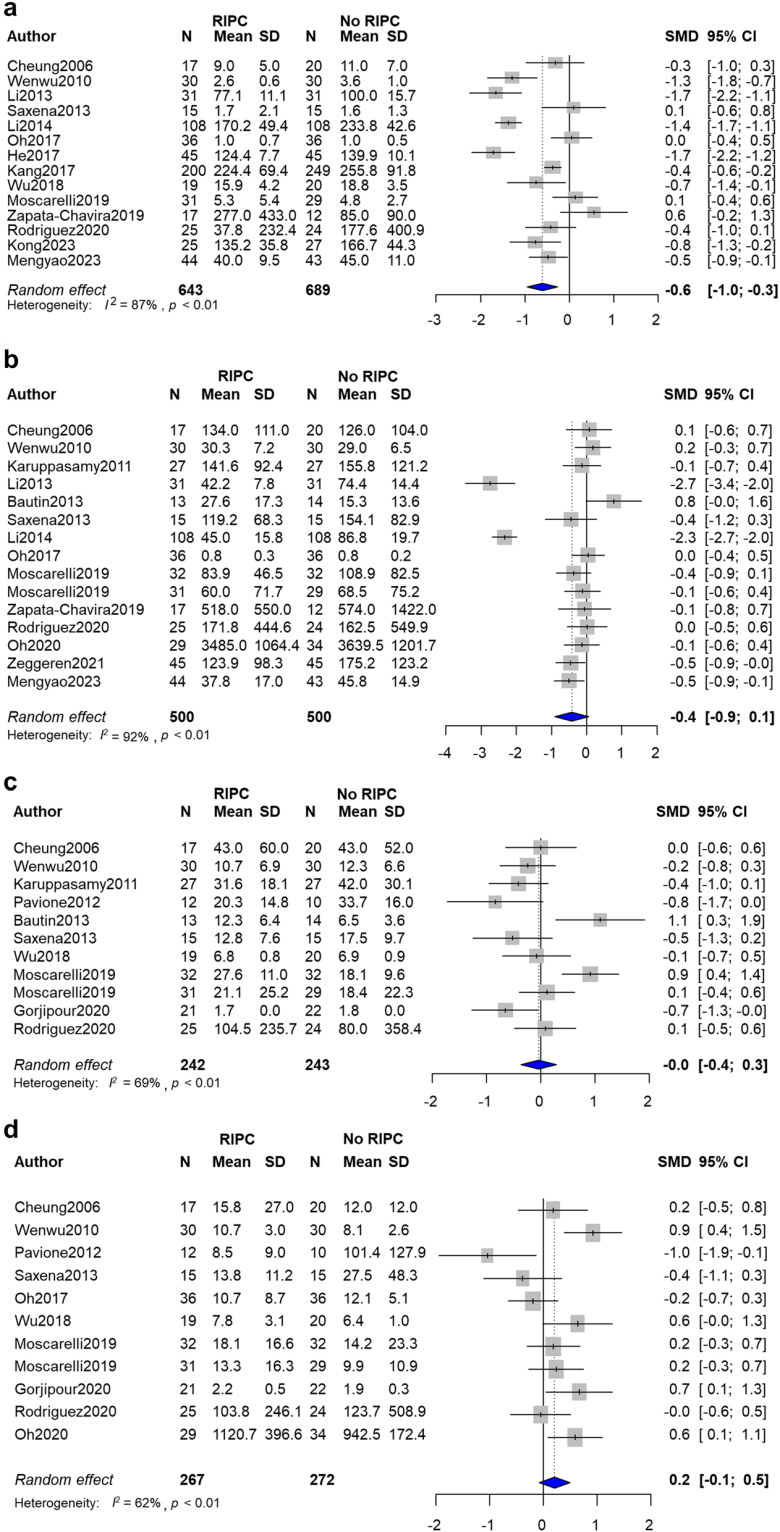
Forest plot with 95% confidence interval (CI) of serum level of tumor necrosis factor α (TNFα) (**a**), interleukin (IL)-6 (**b**), IL-8 (**c**) and IL-10 (**d**). *SD* standard deviation, *SMD* standardised mean difference.

Fourteen trials with 1,332 patients reported the serum TNFα levels at 24 h after surgery^4,9,15,16,18,20,26,27,29,42,46,69–71^. The RIPC group showed lower serum TNFα levels in comparison to the control group with substantial heterogeneity (SMD − 0.6, 95% CI − 1.0 to − 0.3, I^2^ = 87%) (Fig. 4a). A subgroup analysis revealed that the effect of RIPC on the TNFα level was augmented on lung surgery in comparison to other types of surgery (test for subgroup differences, p < 0.01) (Supplemental Fig. S4c). The serum IL-6 level at 24 h after surgery was reported in 14 trials^4,9,16–18,27,29,42,44,46,63,69,71,72^, which did not show statistical significance (SMD − 0.4, 95% CI − 0.9 to 0.1, I^2^ = 92%) (Fig. 4b). The serum IL-8 at 24 h was reported in 10 trials^4,18,20,22,27,38,44,46,69,72^, which was not significantly different (SMD 0.0, 95% CI − 0.4 to 0.3, I^2^ = 69%) (Fig. 4c). Likewise, the serum IL-10 level reported in 10 trials showed similar result (SMD 0.2, 95% CI − 0.1 to 0.5, I^2^ = 62%) (Fig. 4d)^4,18,20,22,27,38,42,46,63,69^.

## Discussion

The present meta-analysis showed a significant benefit of RIPC on oxygenation in patients undergoing various surgical procedures under general anesthesia. Furthermore, we showed that RIPC improved other respiratory outcomes, including A-a DO_2_ and the respiratory index. Finally, we found that RIPC had beneficial effects with respect to the reduced duration of MV after surgery and the lower incidence of postoperative ARDS.

The lung protective effect of RIPC has recently been evaluated in patients undergoing major surgery including aortic aneurysm repair, pulmonary resection and cardiac surgery^8,9,29^. Although the effect after 24 h postoperatively was not analyzed in our study, othe results are in accordance with these studies. However, the effect of RIPC with respect to the improvement of the P/F ratio showed substantial heterogeneity. Past studies showed that RIPC failed to show a beneficial effect in paediatric patients undergoing cardiac surgery^73^. Additionally, the interaction between RIPC and anesthetic agents is controversial^73–76^. Although these factors were examined in a sub-group analysis, we failed to find the cause of heterogeneity, as the differences between the groups were non-significant. With these results, we further performed a *post-hoc* sub-group analysis. Based on the past reports of which cardioprotective effect of RIPC depends on the duration and volume of tissue exposed to ischemia^77,78^, the analysis was according to the place of cuff and total inflation time, which also showed non-significant differences between the groups.

The P/F ratio is a widely used clinical indicator of oxygenation, although it is affected by F_I_O_2_, ventilation perfusion mismatch, and pulmonary shunting. Therefore, we also investigated other oxygenation indices: A-a DO_2_ and the respiratory index. Unlike with the pooled data of the P/F ratio, several trials that included those indices were targeted at pediatric patients^4,18,21,24^. Both A-a DO_2_ and the respiratory index were improved in the RIPC group, which reinforces the effectiveness of RIPC on oxygenation in surgical patients.

Most eligible trials evaluated cardiovascular surgery, which often requires postoperative MV. We further evaluated the effect of RIPC on the duration of MV. Forty-seven trials recorded the duration of postoperative MV. Each of the trials reported no significance difference in the duration of MV between the RIPC and control groups. However, a substantial proportion of studies showed a trend toward shorter MV duration in the RIPC group. Likewise, Min et al. reported that RIPC reduced the incidence of MV > 48 h after cardiac surgery^8^. Similarly to past studies^79,80^, the present analysis showed that RIPC was associated with a slightly shorter duration of MV (0.9 h). Our analysis also showed high heterogeneity (I^2^ = 78%), which was not explained by a sub-group analysis stratified by age, anesthetic regimen, place of inflation cuff or total inflation time. We speculated that the timing of extubation and the duration of MV may be affected by various factors, including recovery from anesthesia or sedatives, haemodynamic/haemostatic stability, and even human resources in the ICU.

We also investigated whether RIPC reduced the incidence of ARDS. Although the management of ARDS—including lung protective ventilation, the avoidance of excess fluid administration, prone positioning, and steroid therapy—have become widely known, ARDS is still a common cause of postoperative lung injury and worsens mortality and morbidity in surgical patients^81,82^. The results of integrated studies reported that RIPC is associated with a lower incidence of ARDS with a risk ratio of 0.73 and low heterogeneity (Fig. 3b). In past studies, the reported incidence of postoperative ARDS ranged from 3.4 to 20%^82,83^, whereas the studies included in our analysis showed a higher incidence of 5.5–54%^9,12,14^. One possible reason for the discrepancy is that the included studies contained patients treated with highly invasive procedures (e.g., cardiac surgery and lung lobectomy). Another possible explanation is that the definition of ARDS has changed with the times as what was once defined as acute lung injury is now included in mild ARDS whose P/F ratio ranges 200–300 Torr^84,85^. Most of included trials focused on ALI defined according to the diagnostic criteria of American-European Consensus Conference 1994, therefore the effect of RIPC on moderate to severe ARDS requires further investigation^12,14,84^.

Although past studies on RIPC have mostly focused on the heart and kidneys, the mechanism of RIPC in lung tissue has also been investigated^86,87^. We further investigated the correlation of inflammatory cytokines and RIPC. In accordance with another recent analysis^11^, our analysis observed that RIPC was associated with a reduced level of serum TNFα with high heterogeneity, which may be due to the difference of anesthetic method or surgery type (Supplemental Fig. S4). It is speculated that volatile anesthetics have an anti-inflammatory effect, which may mask the effect of RIPC^88^. However, the interpretation of the results should be noted because the number of trials with total intravenous anesthesia was small.

The present study was associated with several limitations. First, most of our analyses showed high heterogeneity. Although we conducted a subgroup analysis based on the findings in past studies, the cause of heterogeneity was not found, especially in regard to oxygenation indices. Second, the possibility of publication bias in relation to our primary outcome, the P/F ratio at 24 h, cannot be excluded due to the small number of trials. These are reasons for downgrading the quality level of a body of evidence. Third, some of the included trials showed a high or unclear risk of bias. Although our main result was not changed even when limited to trials with a low risk of bias (Supplemental Fig. S2), well-designed RCTs are needed to verify the conclusions. Fourth, our analyses have several secondary outcomes. Therefore, the risk of type I error inflates due to multiple comparison. Fifth, 58 of the 71 included trials involved cardiac surgery, although we imposed no restriction on surgery type. Therefore, it is unclear whether the reported effects of RIPC are applicable to other types of surgery (e.g., major abdominal surgery). Finally, the lung-protective effect of RIPC was not evaluated by biomarkers that directly reflect lung injury. Although several studies assessed lung oxidative stress markers (e.g., serum malondialdehyde or 8-isoprostane), a large number of the included studies did not measure specific markers^7,9^. Further studies investigating the efficacy of RIPC on lung protective effects, such as the measurement of lung-specific biomarkers or collecting samples from bronchoalveolar lavage fluid, should be conducted. Likewise, studies examining other protective effect of RIPC on non-lung organ such as kidney or brain and its impact on mortality or hospital stay are needed because past studies including systematic review presented inconsistent results or indicated inconclusiveness^89–91^.

In conclusion, the present meta-analysis showed that RIPC improved oxygenation after surgery under general anesthesia. Additionally, RIPC shortened the duration of postoperative MV and reduced the incidence of postoperative ARDS. These findings warrant further investigations focusing on the lung protective effects of RIPC.

## Methods

This meta-analysis was performed according to the PRISMA statement as shown in Fig. 1. The study protocol was registered in the University Hospital Medical Information Network (registration number: UMIN000030918).

### Eligibility criteria

The inclusion criteria for the analysis required that each study be a prospective randomized trial that evaluated the effectiveness of RIPC in patients undergoing surgery with general anesthesia. There were no restrictions on the type of surgery or anesthetic technique. Animal studies, review papers, and non-randomized controlled trials were excluded.

### Search strategy

A comprehensive literature search was performed using MEDLINE, Embase, CENTRAL, Web of Science, Clinical Trials.gov, EU Clinical Trials Registry, and UMIN for randomized controlled trials (RCTs). The following key words were used: remote ischemic preconditioning, anesthesia, and surgical procedures. The full-text of the search formula is shown in Supplemental Document 1. There were no language restrictions. The most recent search was performed in December 2021.

### Data extraction

Two authors (SK and one of CY, AY, DN, or TM) independently assessed the titles and abstracts of the studies identified by the search strategies. We retrieved the full-text versions of potentially relevant studies selected by at least one author, and those that met the inclusion criteria were examined separately. Discrepancies among the authors were resolved by consensus through discussion. Each author also performed data abstraction from the text, tables, or graphs, using standardised data collection forms. Continuous variables were collected in mean ± SD format, and data in median ± IQR format were converted to mean ± SD using the Hozo method. We extracted the baseline characteristics of eligible trials, including first author, year of publication, number of patients, mean age of the patients, type of surgery, and RIPC protocol. Data extracted from each RCT included the following outcomes: (1) indices of oxygenation, including P_a_O_2_/F_I_O_2_ (P/F) ratio, alveolar-arterial oxygen difference (A-a DO_2_), and respiratory index defined as A-a DO_2_/P_a_O_2_ at 24 h after surgery; (2) serum cytokine levels, including interleukin (IL)-6, IL-8, IL-10, and tumor necrosis factor α (TNFα) at 24 h after surgery; and (3) the duration of postoperative mechanical ventilation (MV) and the incidence of postoperative ARDS/acute lung injury (ALI) defined according to the diagnostic criteria of American-European Consensus Conference 1994 or the Berlin definition, depending on the conducted period of each trial^84,85^.

### Risk of bias assessment

We estimated the risk of bias in the following methodological domains: sequence generation, allocation concealment, blinding of participants, blinding of healthcare providers, blinding of data collectors, blinding of outcome assessors, incomplete outcome data, selective outcome reporting, and other potential threats to validity. The risk of bias was assessed as low, high, or unclear in each domain.

### Quality of evidence assessment

We applied the Grading of Recommendations Assessment, Development and Evaluation (GRADE) approach to assess the quality of evidence of the main outcomes as follows: very low, low, moderate, or high.

### Data synthesis and analysis

We used the random-effects model of DerSimonian and Laird and calculated the risk ratio (RR) with 95% confidence interval (CI) for dichotomous data^92^. Pooled effects of RIPC on continuous data were estimated using the mean difference (MD) or standardized mean difference (SMD) with 95% CI. We conducted a sensitivity analysis according to the risk of bias. Heterogeneity across studies was quantified using the I^2^ statistic, which was considered as low, moderate or high if the value exceeded 25%, 50% or 75%. We conducted a predefined subgroup analysis when there is high heterogeneity: patient age (adult or child), anesthetic method (volatile or intravenous anesthesia), and surgery type (lung surgery or not). A forest plot was used to graphically represent the effect of treatment. The small study effect was evaluated using Egger’s regression asymmetry test, with p-values of < 0.1 considered statistically significant. Statistical analyses were performed using the R statistical software package, version 4.0.5 (R Foundation for Statistical computing, Vienna, Austria). The “meta” and “metafor” packages were used to perform the meta-analysis.

### Supplementary Information

Supplementary Information 1.

Supplementary Figure S1.

Supplementary Figure S2.

Supplementary Figure S3.

Supplementary Figure S4.

Supplementary Table S1.

Supplementary Table S2.

## Data Availability

The datasets used and analyzed during the current study are available from the corresponding author on reasonable request.
