# Supplementary material for: Effect of remote ischemic preconditioning on lung function after surgery under general anesthesia: a systematic review and meta-analysis
Source: Sci Rep. 2023 Oct 18;13:17720. doi: 10.1038/s41598-023-44833-w (PMC10584824; doi:10.1038/s41598-023-44833-w)
Supplement: Supplementary file 1 — Supplementary Information 1. [file 41598_2023_44833_MOESM1_ESM.docx]

**Supplemental Document1** The full-text of the research formula

(("anaesthesia"[All Fields] OR "anesthesia"[MeSH Terms] OR "anesthesia"[All Fields]) OR ("anesthetics"[Pharmacological Action] OR "anesthetics"[MeSH Terms] OR "anesthetics"[All Fields] OR "anesthetic"[All Fields]) OR ("anaesthetics"[All Fields] OR "anesthetics"[Pharmacological Action] OR "anesthetics"[MeSH Terms] OR "anesthetics"[All Fields] OR "anesthesiology"[MeSH Terms] OR "anesthesiology"[All Fields]) OR ("surgical procedures, operative"[MeSH Terms] OR ("surgical"[All Fields] AND "procedures"[All Fields] AND "operative"[All Fields]) OR "operative surgical procedures"[All Fields] OR "operation"[All Fields]) OR ("surgery"[Subheading] OR "surgery"[All Fields] OR "operations"[All Fields]) OR ("surgery"[Subheading] OR "surgery"[All Fields] OR "surgical procedures, operative"[MeSH Terms] OR ("surgical"[All Fields] AND "procedures"[All Fields] AND "operative"[All Fields]) OR "operative surgical procedures"[All Fields] OR "surgery"[All Fields] OR "general surgery"[MeSH Terms] OR ("general"[All Fields] AND "surgery"[All Fields]) OR "general surgery"[All Fields]) OR ("surgical procedures, operative"[MeSH Terms] OR ("surgical"[All Fields] AND "procedures"[All Fields] AND "operative"[All Fields]) OR "operative surgical procedures"[All Fields] OR "surgeries"[All Fields])) AND (remote[All Fields] AND (("ischemia"[MeSH Terms] OR "ischemia"[All Fields] OR "ischemic"[All Fields]) OR ("ischemia"[MeSH Terms] OR "ischemia"[All Fields] OR "ischaemic"[All Fields])) AND preconditioning[All Fields]) AND (randomized controlled trial[pt] OR controlled clinical trial[pt] OR randomized[tiab] OR placebo[tiab] OR "drug therapy"[Subheading] OR randomly[tiab] OR trial[tiab] OR groups[tiab]) NOT ("animals"[MeSH Terms] NOT "humans"[MeSH Terms])
