# Supplementary figures and images for: Effect of remote ischemic preconditioning on lung function after surgery under general anesthesia: a systematic review and meta-analysis
Source: Sci Rep. 2023 Oct 18;13:17720. doi: 10.1038/s41598-023-44833-w (PMC10584824; doi:10.1038/s41598-023-44833-w)

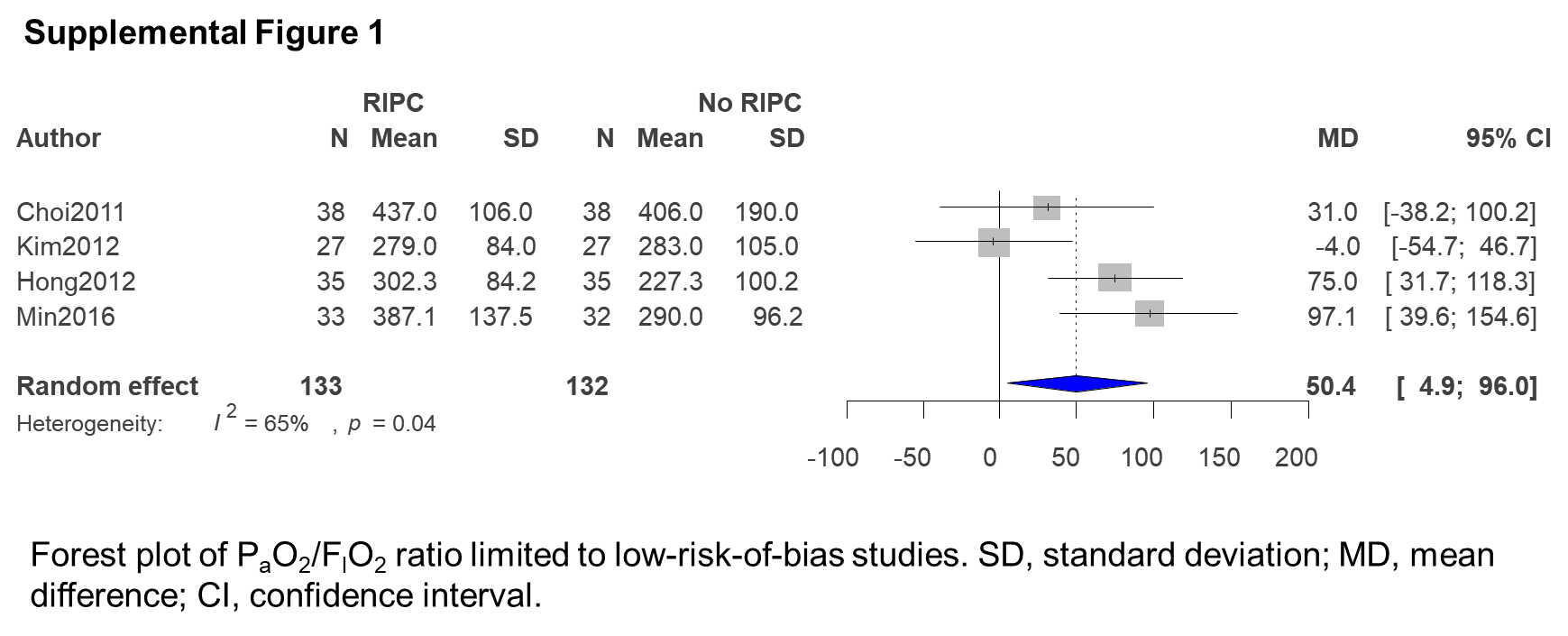

Supplement: Supplementary file 2 — Supplementary Figure S1. [file 41598_2023_44833_MOESM2_ESM.tiff]

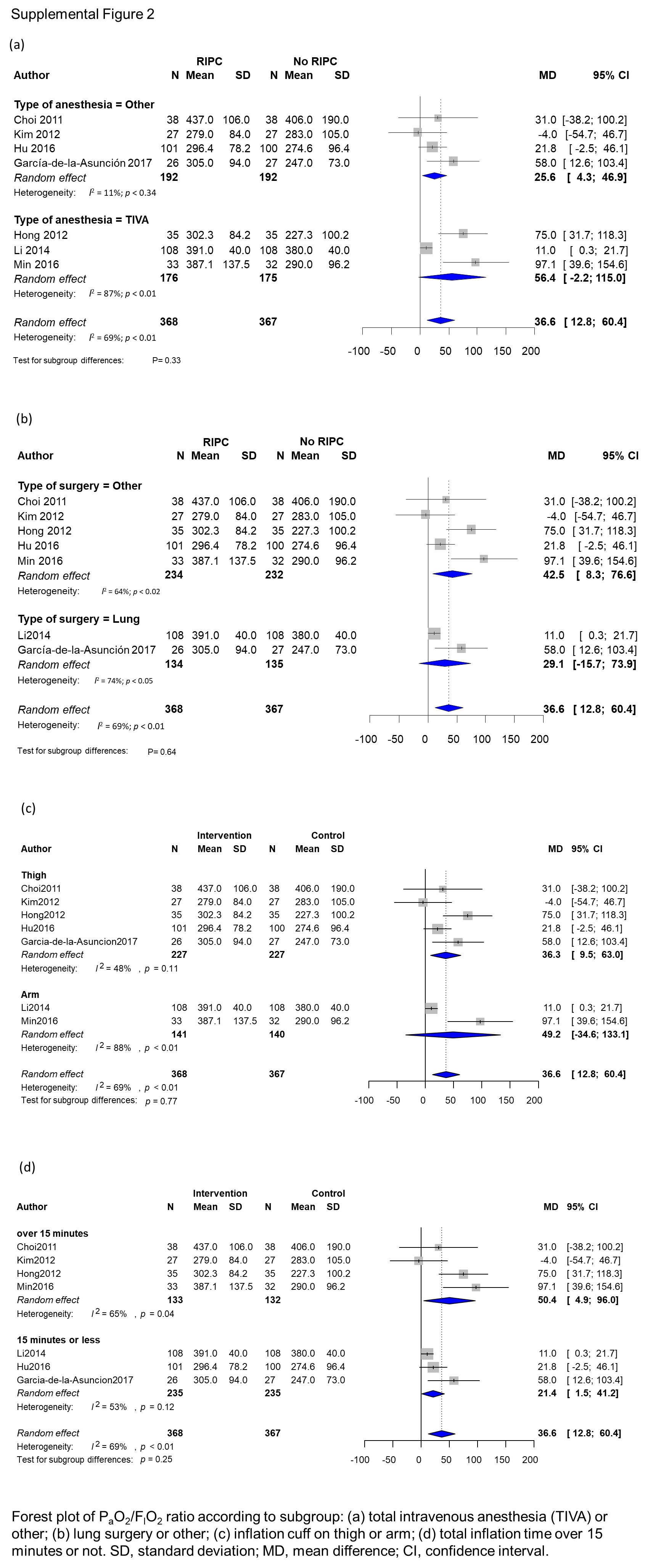

Supplement: Supplementary file 3 — Supplementary Figure S2. [file 41598_2023_44833_MOESM3_ESM.tiff]

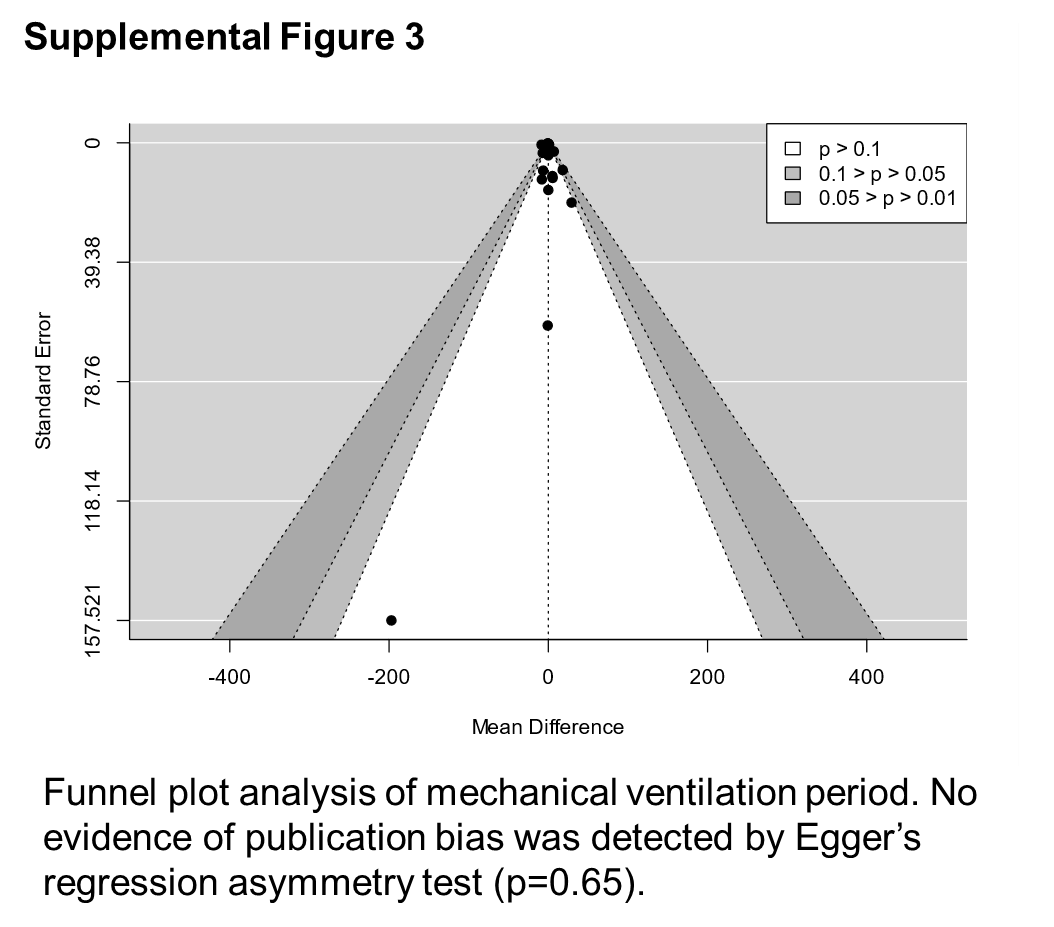

Supplement: Supplementary file 4 — Supplementary Figure S3. [file 41598_2023_44833_MOESM4_ESM.tiff]

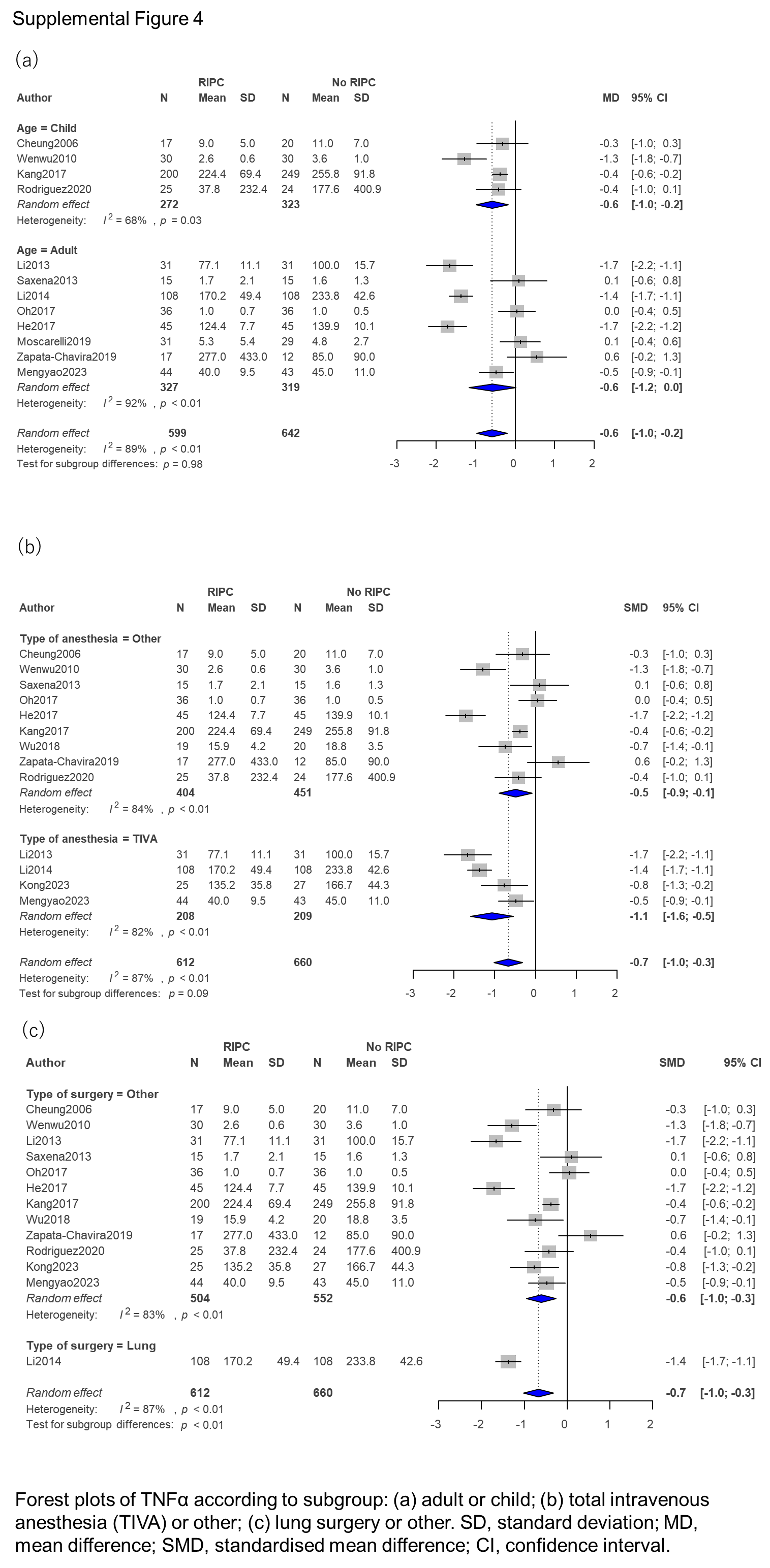

Supplement: Supplementary file 5 — Supplementary Figure S4. [file 41598_2023_44833_MOESM5_ESM.tiff]
