# Supplementary material for: Effect of remote ischemic preconditioning on lung function after surgery under general anesthesia: a systematic review and meta-analysis
Source: Sci Rep. 2023 Oct 18;13:17720. doi: 10.1038/s41598-023-44833-w (PMC10584824; doi:10.1038/s41598-023-44833-w)
Supplement: Supplementary file 6 — Supplementary Table S1. [file 41598_2023_44833_MOESM6_ESM.docx]

**Supplemental Table 1** Characteristics of the included trials

| Trial | Adult/Child | No. of patients (RIPC/Control) | Type of anesthesia | Type of surgery | Timing of RIPC | Duration and cycles of RIPC | Site of inflation cuff | Inflation pressure　(mmHg) |
| --- | --- | --- | --- | --- | --- | --- | --- | --- |
| García-de-la-Asunción 2017 | Adult | 26/27 | Volatile anesthesia | Lung lobectomy | After induction of anesthesia and before start of surgery | 3 cycles of 5-minute ischemia and 5-minute reperfusion | Thigh | 200 |
| Li 2014 | Adult | 108/108 | TIVA | Lung lobectomy | After induction of anesthesia and before start of surgery | 3 cycles of 5-minute ischemia and 5-minute reperfusion | Arm | 200 |
| Li 2013 | Adult | 31/31 | TIVA | Cardiac surgery | After induction of anesthesia and before start of surgery | 3 cycles of 5-minute ischemia and 5-minute reperfusion | Arm | 200 |
| Wenwu 2010 | Child | 30/30 | Not specified or not documented | Cardiac surgery | At 24 h and at 1 h before start of surgery | 3 cycles of 5-minute ischemia and 5-minute reperfusion | Arm | 240 |
| Cheung 2006 | Child | 17/20 | Volatile anesthesia | Cardiac surgery | Before start of CPB | 4 cycles of 5-minute ischemia and 5-minute reperfusion | Thigh | SBP+15 |
|  |  |  |  |  |  |  |  |  |
|  |  |  |  |  |  |  |  |  |
|  |  |  |  |  |  |  |  |  |
| Trial | Adult/Child | No. of patients (RIPC/Control) | Type of anesthesia | Type of surgery | Timing of RIPC | Duration and cycles of RIPC | Site of inflation cuff | Inflation pressure　(mmHg) |
| Luo 2011 | Child | 20/20 | Volatile anesthesia | Cardiac surgery | After induction of anesthesia | 3 cycles of 5-minute ischemia and 5-minute reperfusion | Thigh | 200-300 |
| Wu 2018 | Child | 55/57 | Volatile anesthesia | Cardiac surgery | At 1 h before CPB | 3 cycles of 5-minute ischemia and 5-minute reperfusion | Thigh | SBP+30 |
| Kim 2012 | Adult | 27/27 | Not specified or not documented | Cardiac surgery | After induction of anesthesia | 3 cycles of 10-minute ischemia and 10-minute reperfusion | Thigh | 250 |
| Lee 2012 | Child | 27/28 | Not specified or not documented | Cardiac surgery | After induction of anesthesia | 4 cycles of 5-minute ischemia and 5-minute reperfusion | Thigh | SBP+30 |
| Choi 2011 | Adult | 38/38 | Volatile anesthesia | Cardiac surgery | At least 10 min before start of CPB | 3 cycles of 10-minute ischemia and 10-minute reperfusion | Thigh | 250 |
| Hong 2012 | Adult | 35/35 | TIVA | Cardiac surgery | After induction of anesthesia | 4 cycles of 5-minute ischemia and 5-minute reperfusion | Thigh | 200 |

**Supplemental Table 1** (Continued)

| Trial | Adult/Child | No. of patients (RIPC/Control) | Type of anesthesia | Type of surgery | Timing of RIPC | Duration and cycles of RIPC | Site of inflation cuff | Inflation pressure |
| --- | --- | --- | --- | --- | --- | --- | --- | --- |
| Bautin 2013 | Adult | 13/14 | Not specified or not documented | Cardiac surgery | After induction of anesthesia | 3 cycles of 5-minute ischemia | Thigh | Not documented |
| Oh 2017 | Adult | 36/36 | Volatile anesthesia | Other surgery | After induction of anesthesia | 3 cycles of 5-minute ischemia | Thigh | Twice the SBP |
| Yildirim 2016 | Adult | 30/30 | Not specified or not documented | Cardiac surgery | After induction of anesthesia and just before surgical incision | 3 cycles of 5-minute ischemia and 5-minute reperfusion | Thigh | 200 |
| Pavione 2012 | Child | 12/10 | Volatile anesthesia | Cardiac surgery | Approximately 24 h before cardiac surgery | 4 cycles of 5-minute ischemia and 5-minute reperfusion | Thigh | SBP+15 |
| Hu 2016 | Adult | 101/100 | Not specified or not documented | Cardiac surgery | After cross-clamping of the aorta | 3 cycles of 5-minute ischemia and 5-minute reperfusion | Thigh | 600 |
| García-de-la-Asunción 2011 | Adult | 20/20 | Not specified or not documented | Lung lobectomy | Before surgery | 3 cycles of 5-minute ischemia and 5-minute reperfusion | Arm | Not documented |
| Meybohm 2013 | Adult | 90/90 | TIVA | Cardiac surgery | After induction of anesthesia | 4 cycles of 5-minute ischemia and 5-minute reperfusion | Arm | 200 |
| Trial | Adult/Child | No. of patients (RIPC/Control) | Type of anesthesia | Type of surgery | Timing of RIPC | Duration and cycles of RIPC | Site of inflation cuff | Inflation pressure |
| Min 2016 | Adult | 33/32 | TIVA | Cardiac surgery | Before start of CPB | 4 cycles of 5-minute ischemia and 5-minute reperfusion | Arm | 200 |
| Karuppasamy 2011 | Adult | 27/27 | Volatile anesthesia | Cardiac surgery | After induction of anesthesia | 3 cycles of 5-minute ischemia and 5-minute reperfusion | Arm | 200 |
| Williams 2012 | Adult | 47/48 | Volatile anesthesia | Cardiac surgery | At start of surgery | 3 cycles of 5-minute ischemia and 5-minute reperfusion | Arm | 200 |
| Saxena 2013 | Adult | 15/15 | Not specified or not documented | Cardiac surgery | After induction of anesthesia | 3 cycles of 5-minute ischemia and 5-minute reperfusion | Arm | SBP+20 |
| Lomivorotov 2012 | Adult | 40/40 | Volatile anesthesia | Cardiac surgery | After induction of anesthesia | 3 cycles of 5-minute ischemia and 5-minute reperfusion | Arm | 200 |
| Venugopal 2009 | Adult | 39/41 | Not specified or not documented | Cardiac surgery | Not documented | 3 cycles of 5-minute ischemia and 5-minute reperfusion | Arm | 200 |
| Gallagher 2015 | Adult | 43/43 | Not specified or not documented | Cardiac surgery | After induction of anesthesia | 3 cycles of 5-minute ischemia and 5-minute reperfusion | Arm | SBP+50 |

**Supplemental Table 1** (Continued)

| Trial | Adult/Child | No. of patients (RIPC/Control) | Type of anesthesia | Type of surgery | Timing of RIPC | Duration and cycles of RIPC | Site of inflation cuff | Inflation pressure |
| --- | --- | --- | --- | --- | --- | --- | --- | --- |
| He 2017 | Adult | 45/45 | Volatile anesthesia | Other surgery | 45 min before induction of anesthesia | 3 cycles of 5-minute ischemia and 5-minute reperfusion | Arm | 200 |
| Pinaud 2015 | Adult | 50/49 | Volatile anesthesia | Cardiac surgery | After induction of anesthesia | 3 cycles of 5-minute ischemia and 5-minute reperfusion | Arm | 200 |
| McCrindle 2014 | Child | 148/151 | Volatile anesthesia | Cardiac surgery | After induction of anesthesia | 4 cycles of 5-minute ischemia and 5-minute reperfusion | Thigh | SBP+15 |
| Jones 2013 | Child | 20/19 | Volatile anesthesia | Cardiac surgery | After induction of anesthesia | 4 cycles of 5-minute ischemia and 5-minute reperfusion | Thigh | SBP+15 |
| Rahman 2010 | Adult | 80/82 | Not specified or not documented | Cardiac surgery | After induction of anesthesia | 3 cycles of 5-minute ischemia and 5-minute reperfusion | Arm | 200 |
| Thielmann 2010 | Adult | 27/26 | Not specified or not documented | Cardiac surgery | After induction of anesthesia | 3 cycles of 5-minute ischemia and 5-minute reperfusion | Arm | 200 |
| Holmberg 2014 | Adult | 20/21 | Volatile anesthesia | Cardiac surgery | After induction of anesthesia | 3 cycles of 5-minute ischemia and 5-minute reperfusion | Arm | 200 |
| Nouraei 2016 | Adult | 50/49 | Not specified or not documented | Cardiac surgery | After induction of anesthesia | 3 cycles of 5-minute ischemia and 5-minute reperfusion | Thigh | 200 |
| Hong 2014 | Adult | 644/636 | TIVA | Cardiac surgery | After induction of anesthesia | 4 cycles of 5-minute ischemia and 5-minute reperfusion | Arm | 200 |
| Joung 2013 | Adult | 35/35 | TIVA | Cardiac surgery | Before coronary artery anastomosis | 4 cycles of 5-minute ischemia and 5-minute reperfusion | Arm | 200 |
| Zarbock 2015 | Adult | 120/120 | Volatile anesthesia | Cardiac surgery | After induction of anesthesia | 3 cycles of 5-minute ischemia and 5-minute reperfusion | Arm | 200 |
| Pepe 2013 | Child | 20/20 | Not specified or not documented | Cardiac surgery | After induction of anesthesia | 4 cycles of 5-minute ischemia and 5-minute reperfusion | Thigh | SBP+30 |
| Elgariah 2017 | Adult | 36/37 | Not specified or not documented | Cardiac surgery | After induction of anesthesia | 4 cycles of 5-minute ischemia and 5-minute reperfusion | Arm | 200 |

**Supplemental Table 1** (Continued)

| Trial | Adult/Child | No. of patients (RIPC/Control) | Type of anesthesia | Type of surgery | Timing of RIPC | Duration and cycles of RIPC | Site of inflation cuff | Inflation pressure |
| --- | --- | --- | --- | --- | --- | --- | --- | --- |
| Kim 2017 | Adult | 80/80 | Not specified or not documented | Cardiac surgery | 24 to 48 h before surgery | 4 cycles of 5-minute ischemia and 5-minute reperfusion | Arm | 200 |
| Li 2010 | Adult | 26/27 | Not specified or not documented | Cardiac surgery | After induction of anesthesia | 3 cycles of 4-minute ischemia and 4-minute reperfusion | Thigh | 600 |
| Guerra 2017 | Child | 18/20 | Volatile anesthesia | Cardiac surgery | 24 to 48 h before surgery | 4 cycles of 5-minute ischemia and 5-minute reperfusion | Thigh | SBP+20 |
| Kang 2017 | Child | 200/249 | Not specified or not documented | Cardiac surgery | 12 h before surgery | 4 cycles of 5-minute ischemia and 5-minute reperfusion | Thigh | SBP+30 |
| Young 2012 | Adult | 48/48 | Volatile anesthesia | Cardiac surgery | At start of surgery | 3 cycles of 5-minute ischemia and 5-minute reperfusion | Arm | 200 |
| Thielmann 2013 | Adult | 162/167 | Not specified or not documented | Cardiac surgery | After induction of anesthesia | 3 cycles of 5-minute ischemia and 5-minute reperfusion | Arm | 200 |
| Gedik 2017 | Adult | 23/23 | Volatile anesthesia | Cardiac surgery | After induction of anesthesia | 3 cycles of 5-minute ischemia and 5-minute reperfusion | Arm | 200 |
| Chen 2012 | Adult | 20/20 | Not specified or not documented | Other surgery | Not documented | 3 cycles of 5-minute ischemia and 5-minute reperfusion | Thigh | 300 |
| Wang 2019 | Adult | 33/32 | Volatile anesthesia | Cardiac surgery | Right after end of induction of anesthesia | 4 cycles of 5-minute ischemia and 5-minute reperfusion | Arm | SBP+40 |
| Wang 2019 | Adult | 60/60 | TIVA | Other surgery | After induction of anesthesia | 4 cycles of 5-minute ischemia and 5-minute reperfusion | Arm | 200 |
| Zhou 2019 | Adult | 65/65 | Not specified or not documented | Cardiac surgery | After induction of anesthesia and before surgical incision | 4 cycles of 5-minute ischemia and 5-minute reperfusion | Arm | 200 |
| Jin 2019 | Adult | 121/120 | Not specified or not documented | Cardiac surgery | Not documented | 2 cycles of 5-minute ischemia and 5-minute reperfusion | Arm and Thigh | 200 |
| Moscarelli 2019 | Adult | 63/61 | Volatile anesthesia | Lung lobectomy | Not documented | 4 cycles of 5-minute ischemia and 5-minute reperfusion | Arm | 200 |

**Supplemental Table 1** (Continued)

| Trial | Adult/Child | No. of patients (RIPC/Control) | Type of anesthesia | Type of surgery | Timing of RIPC | Duration and cycles of RIPC | Site of inflation cuff | Inflation pressure |
| --- | --- | --- | --- | --- | --- | --- | --- | --- |
| Yildirim 2018 | Adult | 30/30 | TIVA | Cardiac surgery | After induction of anesthesia | 3 cycles of 5-minute ischemia and 5-minute reperfusion | Thigh | 200 |
| Gasparovic 2019 | Adult | 33/32 | Volatile anesthesia | Cardiac surgery | After induction of anesthesia and prior to surgical incision | 3 cycles of 5-minute ischemia and 5-minute reperfusion | Arm | 200 |
| Jiang 2019 | Adult | 104/102 | TIVA | Cardiac surgery | After induction of anesthesia | 3 cycles of 5-minute ischemia and 5-minute reperfusion | Arm | Not documented |
| Zapata-Chavira 2019 | Adult | 17/12 | Not specified or not documented | Other surgery | Immediately prior to initiation of laparotomy | 1 cycle of 10-minute ischemia | Thigh | 200 |
| Zeggeren 2021 | Adult | 45/45 | Volatile anesthesia | Other surgery | After induction of anesthesia | 3 cycles of 5-minute ischemia and 5-minute reperfusion | Arm | 200 |
| Cho 2020 | Adult | 26/28 | TIVA | Cardiac surgery | After induction of anesthesia | 3 cycles of 5-minute ischemia and 5-minute reperfusion | Arm | 200 |
|  |  |  |  |  |  |  |  |  |
| Trial | Adult/Child | No. of patients (RIPC/Control) | Type of anesthesia | Type of surgery | Timing of RIPC | Duration and cycles of RIPC | Site of inflation cuff | Inflation pressure |
| Gorjipour 2020 | Adult | 21/22 | TIVA | Cardiac surgery | Not documented | 3 cycles of 5-minute ischemia and 5-minute reperfusion | Arm | SBP+15-20 |
| Kim 2020 | Adult | 28/28 | TIVA | Cardiac surgery | After induction of anesthesia | 4 cycles of 5-minute ischemia and 5-minute reperfusion | Arm | 200 |
| Miličić 2020 | Adult | 20/22 | Volatile anesthesia | Cardiac surgery | After induction of anesthesia | 3 cycles of 5-minute ischemia and 5-minute reperfusion | Arm | 200 |
| Rodriguez 2020 | Child | 25/24 | Volatile anesthesia | Cardiac surgery | 15 to 20 h before surgery and following induction of anesthesia | 3 cycles of 5-minute ischemia and 5-minute reperfusion | Arm (BW>10kg) or Thigh (BW<10kg) | SBP+20 |
| Li 2021 | Adult | 25/25 | Volatile anesthesia | Cardiac surgery | Before CPB | 3 cycles of 5-minute ischemia and 5-minute reperfusion | Thigh | 300 |
| Tosun 2021 | Adult | 10/11 | Volatile anesthesia | Other surgery | Before onset of the anhepatic phase | 3 cycles of 3-minute ischemia and 3-minute reperfusion | Thigh | 250 |
|  |  |  |  |  |  |  |  |  |
| Trial | Adult/Child | No. of patients (RIPC/Control) | Type of anesthesia | Type of surgery | Timing of RIPC | Duration and cycles of RIPC | Site of inflation cuff | Inflation pressure |
| Oh 2020 | Adult | 29/24 | Not specified or not documented | Other surgery | After induction of anesthesia | 3 cycles of 5-minute ischemia and 2-minute reperfusion | Thigh | Twice the SBP |
| Yuansong 2021 | Adult | 20/20 | Not specified or not documented | Cardiac surgery | Before the start of surgery | 3 cycles of 5-minute ischemia and 5-minute reperfusion | Arm | 200 |
| Mehrabanian 2023 | Adult | 25/25 | TIVA | Cardiac surgery | Not documented | 4 cycles of 5-minute ischemia and 5-minute reperfusion | Arm | SBP+15-20 |
| Chiari 2023 | Adult | 104/103 | Volatile anesthesia | Cardiac surgery | After induction of anesthesia | 3 cycles of 5-minute ischemia and 5-minute reperfusion | Arm | 200 |
| Qi 2021 | Child | 51/55 | Volatile anesthesia | Other surgery | After induction of anesthesia | 3 cycles of 5-minute ischemia and 5-minute reperfusion | Thigh | SBP+150 |
| Chun Tian 2023 | Adult | 40/40 | Volatile anesthesia | Other surgery | After induction of anesthesia | 3 cycles of 5-minute ischemia and 5-minute reperfusion | Arm | 200 |
|  |  |  |  |  |  |  |  |  |
| Trial | Adult/Child | No. of patients (RIPC/Control) | Type of anesthesia | Type of surgery | Timing of RIPC | Duration and cycles of RIPC | Site of inflation cuff | Inflation pressure |
| Kong 2023 | Adult | 25/27 | TIVA | Other surgery | After induction of anesthesia | 3 cycles of 5-minute ischemia and 5-minute reperfusion | Arm | 200 |
| Mengyao 2023 | Adult | 44/43 | TIVA | Other surgery | After induction of anesthesia | 3 cycles of 5-minute ischemia and 5-minute reperfusion | Arm | 200 |

RIPC, remote ischemic preconditioning; TIVA, total intravenous anesthesia; CPB, cardiopulmonary bypass; SBP, systolic blood puressure.
