# Supplementary material for: Effect of remote ischemic preconditioning on lung function after surgery under general anesthesia: a systematic review and meta-analysis
Source: Sci Rep. 2023 Oct 18;13:17720. doi: 10.1038/s41598-023-44833-w (PMC10584824; doi:10.1038/s41598-023-44833-w)
Supplement: Supplementary file 7 — Supplementary Table S2. [file 41598_2023_44833_MOESM7_ESM.docx]

**Supplemental Table 2** Risk of bias assessment of the randomized studies included in this meta-analysis.

| Trial | Random sequence generation | Allocation concealment | Patients blinded | Healthcare providers blinded | Data collectors blinded | Outcome assessors blinded | Incomplete outcome data | Selective reporting | Other bias | Summary |
| --- | --- | --- | --- | --- | --- | --- | --- | --- | --- | --- |
| García-de-la-Asunción 2017 | Unclear | Unclear | Low | Low | Low | Low | Low | Low | Low | Unclear |
| Li 2014 | Low | Low | Low | High | Low | Low | Low | Low | Low | High |
| Li 2013 | Low | Low | Low | Low | Low | Low | Low | Low | Low | Low |
| Wenwu 2010 | Unclear | Low | Low | Low | Low | Low | Low | Unclear | Low | Unclear |
| Cheung 2006 | Unclear | Low | Low | Unclear | Unclear | Unclear | Unclear | Unclear | Low | Unclear |
| Luo 2011 | Low | Low | Low | Low | Low | Low | Unclear | Unclear | Unclear | Low |
| Wu 2018 | Low | Low | Low | Low | Low | Low | Low | Low | Low | Low |
| Kim 2012 | Low | Low | Low | Low | Low | Low | Low | Low | Low | Low |
| Lee 2012 | Unclear | Unclear | Unclear | Unclear | Unclear | Unclear | Low | Low | Unclear | Unclear |
| Choi 2011 | Low | Unclear | Low | Low | Unclear | Unclear | Low | Low | Low | Low |
| Hong 2012 | Low | Low | Low | Low | Unclear | Unclear | Low | Low | Low | Low |
| Bautin 2013 | Unclear | Unclear | Unclear | Unclear | Unclear | Unclear | Unclear | Unclear | Unclear | Unclear |
| Oh 2017 | Unclear | Low | Low | Low | Low | Low | Low | Low | Low | Unclear |
| Yildirim 2016 | Unclear | Unclear | Low | Low | Low | Low | Low | Unclear | Unclear | Low |
| Pavione 2012 | Low | Unclear | High | Low | Low | Low | Low | Low | Unclear | Unclear |
| Hu 2016 | Unclear | Unclear | Low | Low | Low | Low | Low | Low | Low | Unclear |
| García-de-la-Asunción 2011 | Unclear | Unclear | Unclear | Unclear | Unclear | Unclear | Unclear | Unclear | Unclear | Unclear |

**Supplemental Table 2** (Continued)

| Trial | Random sequence generation | Allocation concealment | Patients blinded | Healthcare providers blinded | Data collectors blinded | Outcome assessors blinded | Incomplete outcome data | Selective reporting | Other bias | Summary |
| --- | --- | --- | --- | --- | --- | --- | --- | --- | --- | --- |
| Meybohm 2013 | Low | Low | Low | Low | Low | Low | Low | Low | Low | Low |
| Min 2016 | Low | Low | Low | Low | Low | Low | Low | Low | Low | Low |
| Karuppasamy 2011 | Unclear | Low | Low | Low | Low | Low | Low | Low | Low | Unclear |
| Williams 2012 | Low | Low | Low | Low | Low | Low | Low | Low | Low | Low |
| Saxena 2013 | Unclear | Unclear | Low | Low | Unclear | Unclear | Low | Low | Low | Low |
| Lomivorotov 2012 | Unclear | Unclear | Low | Unclear | Unclear | Unclear | Unclear | Unclear | Unclear | Unclear |
| Venugopal 2009 | Unclear | Unclear | Unclear | Unclear | Unclear | Unclear | Unclear | Unclear | Unclear | Unclear |
| Gallagher 2015 | Unclear | Unclear | Low | Low | High | High | Low | Low | Low | Unclear |
| He 2017 | Low | Low | Low | Low | Low | Low | Low | Unclear | Unclear | Low |
| Pinaud 2015 | Low | Unclear | Low | Unclear | Unclear | Unclear | Low | Low | Unclear | Unclear |
| McCrindle 2014 | Low | Low | Low | Low | Low | Low | Low | Unclear | Low | Low |
| Jones 2013 | Low | Low | Low | Low | Low | Low | Low | Low | Low | Low |
| Rahman 2010 | Low | Low | Low | Low | Low | Low | Low | Low | Low | Low |
| Thielmann 2010 | Unclear | Unclear | Low | High | Unclear | Unclear | Low | Low | Low | Unclear |
| Holmberg 2014 | Low | Low | Low | High | Unclear | Unclear | Low | Low | Low | High |

**Supplemental Table 2** (Continued)

| Trial | Random sequence generation | Allocation concealment | Patients blinded | Healthcare providers blinded | Data collectors blinded | Outcome assessors blinded | Incomplete outcome data | Selective reporting | Other bias | Summary |
| --- | --- | --- | --- | --- | --- | --- | --- | --- | --- | --- |
| Nouraei 2016 | Unclear | Unclear | Low | Low | Low | Low | Low | Low | Low | Unclear |
| Hong 2014 | Low | Low | Low | Low | Low | Low | Low | Low | Low | Low |
| Joung 2013 | Low | Unclear | Low | Unclear | Low | Low | Low | Low | Low | Unclear |
| Zarbock 2015 | Low | Low | Low | Low | Low | Low | Low | Low | Low | Low |
| Pepe 2013 | Unclear | Unclear | Low | Unclear | Unclear | Unclear | Low | Low | Low | Unclear |
| Elgariah 2017 | Unclear | Unclear | Unclear | Unclear | Unclear | Unclear | Unclear | Unclear | Unclear | Unclear |
| Kim 2017 | Low | Unclear | Low | Low | Low | Low | Low | Low | Low | Unclear |
| Guerra 2017 | Low | Low | Low | Low | Low | Low | Low | Low | Low | Low |
| Kang 2017 | Low | Low | Low | Low | Low | Low | Low | Unclear | Low | Low |
| Li 2010 | Low | Unclear | Low | Low | Low | Low | Low | Low | Low | Unclear |
| Young 2012 | Low | Low | Low | Low | Low | Low | Low | Low | Low | Low |
| Thielmann 2013 | Low | Low | Low | Low | Low | Low | Low | Low | Low | Low |
| Gedik 2017 | Unclear | Unclear | Unclear | Unclear | Unclear | Unclear | Low | Low | Unclear | Unclear |
| Chen 2012 | Unclear | Unclear | Low | Unclear | Unclear | Unclear | Low | Low | Low | Low |
| Wang 2019 | Low | Unclear | Low | Low | Unclear | Unclear | Unclear | High | High | High |
| Wang 2019 | Low | Low | Low | Low | Low | Low | Low | Low | Low | Low |
| Zhou 2019 | Low | Low | Low | Low | Low | Low | Low | Low | Low | Low |
| Jin 2019 | Unclear | Unclear | Low | Unclear | Low | Low | Low | Low | Low | Unclear |
|  |  |  |  |  |  |  |  |  |  |  |

**Supplemental Table 2** (Continued)

| Trial | Random sequence generation | Allocation concealment | Patients blinded | Healthcare providers blinded | Data collectors blinded | Outcome assessors blinded | Incomplete outcome data | Selective reporting | Other bias | Summary |
| --- | --- | --- | --- | --- | --- | --- | --- | --- | --- | --- |
| Moscarelli 2019 | Low | Low | Low | Low | Low | Low | Low | Low | Low | Low |
| Yildirim 2018 | Unclear | Unclear | Low | Low | Low | Low | Low | Low | Low | Unclear |
| Gasparovic 2019 | Low | Low | Low | Unclear | Low | Low | Low | Low | Low | Low |
| Jiang 2019 | Low | Unclear | Low | Unclear | Unclear | Unclear | Low | Low | Low | Unclear |
| Zapata-Chavira 2019 | Low | Low | Low | Unclear | Unclear | Unclear | Unclear | Unclear | Unclear | Unclear |
| Zeggeren 2021 | Low | Low | Low | Unclear | Low | Low | Low | Low | Low | Unclear |
| Cho 2020 | Low | Low | Low | Low | Low | Low | Low | Low | Low | Low |
| Gorjipour 2020 | Unclear | Unclear | Unclear | Unclear | Unclear | Unclear | Low | Low | Low | Unclear |
| Kim 2020 | Low | Low | Low | Low | Low | Low | Low | Low | Low | Low |
| Miličić 2020 | Low | Low | Low | High | Low | Low | Low | Low | Low | High |
| Rodriguez 2020 | Low | Unclear | Low | High | Low | Low | Low | Low | Low | High |
| Li 2021 | Low | Low | Low | Low | Low | Low | Low | Low | Low | Low |
| Tosun 2021 | Unclear | Unclear | Low | Low | Low | Low | Low | Low | Low | Unclear |
| Oh 2020 | Low | Low | Low | Low | Low | Low | Low | Low | Low | Low |
| Yuansong 2023 | Unclear | Unclear | Unclear | Unclear | Unclear | Unclear | Unclear | Unclear | Unclear | Unclear |
| Mehrabanian 2023 | Low | Unclear | Low | High | Low | Low | Low | Low | Low | High |
| Chiari 2023 | Low | Low | Low | High | High | High | Low | Low | Low | High |
| Qi 2021 | Low | Low | Unclear | Unclear | Unclear | Unclear | Low | Low | Low | Unclear |
| Chun Tian 2023 | Unclear | Unclear | Low | Low | Unclear | Unclear | Low | Low | Low | Unclear |
| Kong 2023 | Unclear | Low | Low | High | Low | Low | Low | Low | Low | Low |
| Mengyao 2023 | Low | Low | Low | Low | Low | Low | Low | Low | Low | Low |
